# Postnatal care could be the key to improving the continuum of care in maternal and child health in Ratanakiri, Cambodia

**DOI:** 10.1371/journal.pone.0198829

**Published:** 2018-06-11

**Authors:** Kimiyo Kikuchi, Junko Yasuoka, Keiko Nanishi, Ashir Ahmed, Yasunobu Nohara, Mariko Nishikitani, Fumihiko Yokota, Tetsuya Mizutani, Naoki Nakashima

**Affiliations:** 1 Institute of Decision Science for a Sustainable Society, Kyushu University, Fukuoka, Japan; 2 Research and Education Center for Prevention of Global Infectious Diseases of Animals, Tokyo University of Agriculture and Technology, Tokyo, Japan; 3 Office of International Academic Affairs, Graduate School of Medicine, the University of Tokyo, Tokyo, Japan; 4 Department of Advanced Information Technology, Kyushu University, Fukuoka, Japan; 5 Medical Information Center, Kyushu University Hospital, Fukuoka, Japan; Tulane University School of Public Health and Tropical Medicine, UNITED STATES

## Abstract

In South-East Asia, the maternal and child mortality rate has declined over the past decades; however, it varies among and within the countries in the region, including Cambodia. The continuum of care is an integrated series of care that women and children are required to avail continuously from pregnancy to the child/motherhood period. This study aimed to assess the completion rate of the continuum of care and examine the factors associated with the continuum of care in Ratanakiri, Cambodia. A cross-sectional study was conducted in Ratanakiri. Overall, 377 women were included, and data were collected via face-to-face interviews using a semi-structured questionnaire. Among them, 5.0% completed the continuum of care (antenatal care at least four times, delivery by skilled birth attendant, and postnatal care at least once). Meanwhile, 18.8% did not receive any care during pregnancy, delivery, and after birth. The highest discontinuation rate was at the postnatal care stage (73.6%). Not receiving any perinatal care was associated with neonatal complications at 6 weeks after birth (adjusted odds ratio [AOR]: 3.075; 95% confidence interval [CI]: 1.310–7.215). Furthermore, a long distance to the health center was negatively associated with completion of the continuum of care (AOR: 0.877; 95% CI: 0.791–0.972). This study indicates the need for efforts to reduce the number of women who discontinue from the continuum of care, as well as who do not receive any care to avoid neonatal complications. Since the discontinuation rate was highest at the postnatal care, postnatal care needs to be promoted more through the antenatal care and delivery services. Furthermore, given that long distance to health facilities was a barrier for receiving the care continuously, our findings suggest the need for a village-based health care system that can provide the basic continuum of care in remote areas.

## Introduction

Maternal and child health has greatly improved owing to the United Nation’s Millennium Development Goals. However, the worldwide incidence of preventable deaths is still high. Approximately 303,000 women die of maternal causes annually [1]. Meanwhile, 5.6 million children under 5 years of age die [1], with 40% of them dying within their first 28 days of life or during the neonatal period [2]. Most of these deaths are preventable [3, 4]. As such, the United Nations has called to end preventable deaths among mothers and children by 2030 in its Sustainable Development Goals [5]. In South-East Asia, the rate of maternal and child mortality has declined over the past decades; however, the rate varies among and within the countries in the region [6].

The concept of the continuum of care has been advocated to improve the status of maternal and child health in the field of global health [7]. The continuum of care is an integrated series of care composed by time and space dimensions that women and children are required to avail continuously [8]. A similar term, “continuity of care”, has been often used in the context of nursing care which represents the consistent approach of care by a single professional [9, 10]. However, the continuum of care emphasizes more on the delivery of required services by different providers in coherent and timely manners [9]. In the concept of the continuum of care, the time dimension is composed of service delivery during adolescence/pre-pregnancy, pregnancy, delivery, postpartum, childhood, and motherhood. At least four antenatal care visits are recommended [11] and recently eight antenatal care visits were recommended to reduce perinatal mortality and women’s experience of care [12]. The established timing for postnatal care visits is within 48 hours, 6–7 days, and at 6 weeks after birth [13]. Meanwhile, the space dimension is composed by three care stages, namely, clinical care, outreach–outpatient care, and family–community care [13]. One study reported that the implementating all care from antenatal to postnatal periods can reduce neonatal mortality by 36–67% [14]. A meta-analysis demonstrated that receiving care from antenatal to postnatal periods may reduce the risk of combined neonatal, perinatal, and maternal mortality by 15% [15]. Another meta-analysis showed that continuous pre-pregnancy and pregnancy stages of care may reduce neonatal and perinatal mortality risk by 21% and 16%, respectively [16]. The continuum of care is an effective strategy to improve maternal and child health. However, it has not been adequately implemented in low- and middle-income countries [8].

Maternal and child mortality has remarkably decreased in Cambodia [1]. The country’s Millennium Development Goals for maternal death and child mortality (goals 4 and 5) were attained five years prior to 2015 [17]. In 2012, the maternal mortality rate was 170 per 100,000 live births, and the under-five mortality rate was 38 per 1,000 live births [18], which was more than 60% and 80% lower compared to that in 2000, respectively. This can be attributed to the expanded coverage of maternal and child health care at the national level: antenatal care (95%), delivery (83%), and postnatal care (90%) [19]. However, regarding the continuum of care, only 60% of women completed the necessary stages of care from pregnancy to the postnatal period [20]. Furthermore, the coverage of care varied among regions [21]. The rate of delivery assisted by skilled birth attendants and postnatal care ranged from 52% to 98% and from 39% to 100%, respectively [19].

To improve the rate of adherence to the continuum of care, factors influencing women’s avail of care or health worker’s service delivery of care in which barriers or discontinuations occur must be identified. Many studies have been conducted to explore the factors associated with antenatal care, skilled birth delivery, or postnatal care [22–26]. Only a few studies, however, have been conducted on factors associated with the completion of the continuum of care as well as on identification of where the discontinuation occurs. Additionally, to encourage both mothers and health workers to prevent health problem, the influence of the discontinuation from the continuum of care on the newborn’s health must be assessed.

Thus, this study aimed to assess the completion rate of the continuum of care and examine the factors associated with the continuum of care in Ratanakiri, Cambodia. The study also examined the association between the mother’s discontinuation stages from the continuum of care and the newborn’s health status.

## Methods

### Study design and setting

This was a cross-sectional study conducted in Ratanakiri Province, Cambodia in December 2015. Ratanakiri Province is located at the northeast border of Cambodia; it borders both Laos and Vietnam. Most of the area is forested with multiple hills, mountains, plateaus, and watersheds. The health status indicators in Ratanakiri are lower than those in other regions of Cambodia. The rate of neonatal mortality was 36/1000 births in 2014, which is higher than the national rate [19]. Overall, 24% of women completed the antenatal and postnatal care and delivered with assistance from a skilled birth attendant according to the combined report from Ratanakiri and Mondulkiri provinces; this rate is one of the lowest among the various provinces in Cambodia [20].

Among 27 health centers in Ratanakiri, seven were included in the study based on their accessibility throughout the year. This was done to avoid the failure of follow-up in the intervention phase, which is planned after this survey. The study participants were selected from the catchment areas of the selected health centers.

### Study population

The study population comprised women who gave birth within 2 years of the date of data collection. In total, 388 women were recruited. The inclusion criterion was residence in the catchment area for any of the seven health centers. Sample size or power computations were not performed due to the lack of information regarding the number of mothers with children under two years old. Therefore, recruitment was performed to include as many mothers as possible in the study. However, a post hoc power analysis was conducted and the sample size was determined to easily achieve a power of greater than 80% using the effect size we observed.

### Operational definitions

In this study, the continuum of care was defined as antenatal care at least four times, delivery by a skilled birth attendant (doctors, midwives or nurses in health center or hospital), and postnatal care at least once. Participants who received all these components, were considered to have completed the continuum of care. The number of women who received postnatal care in this study area was limited; thus, receiving postnatal care at least once was defined as having received postnatal care.

### Data collection

Data were collected via face-to-face interviews and a Geographic Information System in December 2015. The interviews were conducted using a semi-structured questionnaire comprising different components, including questions on the participant’s sociodemographic characteristics, their received maternal and child health care services, and knowledge and behavior regarding health services. Neonatal complications were assessed according to the development of health problems, such as fever, diarrhea, skin pustules, cough, vomiting, excessive crying, and not gaining weight, within 6 weeks of age. The questionnaire was developed in English based on the questionnaire validated in different studies [19, 27–32] and was translated into Khmer by a Cambodian health expert and then validated by another Khmer-speaking health expert for accuracy. Prior to data collection, the surveyors underwent a 1-day orientation on the contents of the questionnaire and how to conduct the interviews.

Global Positioning System data were collected to measure the mother’s actual travel distance from her village to the health center. Data collectors recorded latitude, longitude, altitude, and time, every five seconds during each trip. The actual travel distance was calculated using Arc Map 10.4.

### Data analysis

First, descriptive analysis was performed to explore the characteristics of the women by stratifying them to different categories. Categories were set following the degree of care stages women completed along with the time dimension of the continuum of care (antenatal care, delivery by a skilled birth attendant, and postnatal care): a) women who completed all stages of the continuum of care, b) women who discontinued the continuum of care at the postnatal care stage (those who received antenatal care at least four times and skilled birth attendant care, but did not receive postnatal care), c) women who discontinued the continuum of care at the delivery stage (those who received antenatal care at least four times but did not receive skilled birth attendant or postnatal care), and d) women who discontinued the continuum of care at the antenatal care (those who did not receive any care).

Second, to determine the factors associated with each category, bivariate analyses were conducted. Each category was compared with the counter category (e.g. women who discontinued the continuum of care at the postnatal care stage vs. those who did not discontinue at the postnatal care stage). Third, multiple logistic regression analyses were conducted for each category. Women’s age; literacy; husband’s education; occupation; wealth quartiles; number of children; and child’s age, sex, and neonatal complications were included as covariates following bivariate analyses. We also entered additional variables associated with antenatal, delivery, or postnatal care in previous studies, such as the child’s sex, age, distance from village to health center, knowledge on antenatal care, facility delivery, and postnatal care, as covariates. However, variables that had a high correlation in multi-collinearity analysis or that had less than five responses were not entered into the model. Statistical significance was set at p<0.05 (two-tailed). Data entry and analysis were executed using SPSS version 24 (IBM Co., New York).

### Ethical considerations

Ethical approval was obtained from the National Ethics Committee for Health Research, the Ministry of Health, Cambodia (368 NECHR), Tokyo University of Agriculture and Technology (28–45), the Research Ethics Committee of the University of Tokyo (11030), and the Research Ethics Committee of Kyushu University (1155). Written informed consent was obtained from all participants prior to data collection. Participation was voluntary, and confidentiality was maintained.

## Results

### Overall characteristics of the participants

Among the 388 women who agreed to participate in the survey, 11 were excluded due to missing data. Therefore, 377 women were included in the analyses. The characteristics of the participants are presented in Table 1. The mean age of participants was 25.6 years (standard deviation [SD] 7.8). The majority of women were unable to read (68.2%). The husbands of 45.1% of the participants never received formal education. The majority (91.3%) of the participants were farmers. The participants comprised multiple ethnic groups, such as Khmer (11.2%), Thompoun (23.4%), Charay (20.2%), and Kreung (15.4%).

**Table 1:** Characteristics of the study participants.

Antenatal care at least four times	Overall		Yes			Yes			Yes			No		
Skilled birth attendance			Yes			Yes			No			No		
Postnatal care at least once			Yes			No.			No			No		
	(n = 377)		(n = 19)			(n = 53)			(n = 41)			(n = 71)		
	No.	(%)	No.	(%)	p-value	No.	(%)	p-value	No.	(%)	p-value	No.	(%)	p-value
Age (years)					0.36			0.28			0.05			0.48
<20	80	21.2	2	10.5		7	13.2		11	26.8		13	18.3	
20–29	192	50.9	13	68.4		35	66.0		17	41.5		34	47.9	
30–39	89	23.7	4	21.1		11	20.8		12	29.3		19	26.8	
40+	16	4.2	0	0.0		0	0.0		1	2.4		5	7.0	
Literacy					<0.01			0.04			<0.01			<0.01
Not able to read	257	68.2	7	36.8		22	41.5		31	75.6		64	90.1	
Able to read	120	31.8	12	63.2		31	58.5		10	24.4		7	9.9	
Husband’s education					0.03			0.14			<0.01			<0.01
No education	170	45.1	3	15.8		12	22.6		18	43.9		53	74.6	
Official education	207	54.9	16	84.2		41	77.4		23	56.1		18	25.3	
Socioeconomic status (wealth quintiles)					0.20			0.09			0.15			0.13
Low or lower middle class	189	50.1	10	52.6		24	45.3		18	43.9		42	59.2	
Upper middle or high class	188	49.8	9	47.3		29	54.7		23	56.1		29	42.9	
Ethnicity					<0.01			0.12			0.15			<0.01
Khmer	42	11.2	6	31.6		17	32.1		4	9.8		0	0.0	
Thompoun	88	23.4	1	5.3		12	22.6		9	22.0		15	21.1	
Charay	76	20.2	7	36.8		6	11.3		6	14.6		28	39.4	
Kreung	58	15.4	1	5.3		7	13.2		9	22.0		8	11.3	
Other	113	29.8	4	21.1		11	20.8		13	31.7		20	28.2	
Number of children					0.28			0.13			0.02			0.04
1	140	37.2	6	31.6		24	45.3		12	29.3		16	22.5	
2	112	29.7	9	47.4		20	37.7		12	29.3		25	35.2	
3–4	99	26.2	4	21.1		8	15.1		12	29.3		24	33.8	
5 +	26	6.9	0	0.0		1	1.9		5	7.9		6	8.5	
Childs’ age (month)					0.46			0.95			0.83			0.16
0–5	141	37.4	4	21.1		21	39.6		16	39.0		28	39.4	
6–11	96	25.5	7	36.8		15	28.3		13	31.7		12	16.9	
12–17	73	19.3	4	21.1		7	13.2		4	9.8		19	26.8	
18–23	67	17.8	4	21.1		19	18.9		8	19.5		12	16.9	
Childs’ gender (female)	181	48.0	10	52.6	0.68	28	52.8	0.88	22	53.7	0.80	32	45.1	0.58
Child had health problem within 6 weeks after birth (yes)	97	25.7	5	31.3	0.70	9	18.4	0.03	19	46.3	<0.01	12	17.6	0.05
Actual distance to the health center (km)					<0.01			0.79			0.03			<0.01
< 5.0	42	11.1	9	47.4		7	13.2		2	4.9		11	15.5	
5.0–9.9	98	26.0	6	31.6		21	39.6		13	31.7		10	14.1	
10.0–14.9	132	35.0	2	10.5		18	34.0		15	36.6		18	25.4	
15.0 +	105	27.9	2	10.5		7	13.2		11	26.8		32	45.1	
Knowledge														
Know that antenatal care is recommended	278	73.7	18	94.7	0.03	49	92.5	0.93	39	95.1	0.46	23	32.4	<0.01
Know that facility delivery is recommended	319	84.6	18	94.7	0.21	48	90.6	0.87	37	90.2	0.82	39	54.9	<0.01
Know that postnatal care is recommended	63	16.7	14	73.7	<0.01	3	5.7	<0.01	6	14.6	0.17	4	5.6	<0.01

Overall, 37.2% of the mothers had only one child, and 37.4% had a child aged less than 6 months on the day of the interview (mean 9.37 months, SD 6.88). Nearly half (48.0%) of the mothers’ children were females. Approximately 25.7% of the women had a child who developed a health complication within 6 weeks after birth. Most of the participants (62.9%) lived in the village that was more than 10 km away from the nearest health center (mean distance 13.6 km, SD 8.31). In total, 73.7%, 84.6%, and 16.7% of women were aware that antenatal care, delivery in a healthcare facility, and postnatal care were recommended, respectively.

### Continuum of care

**Fig 1:**
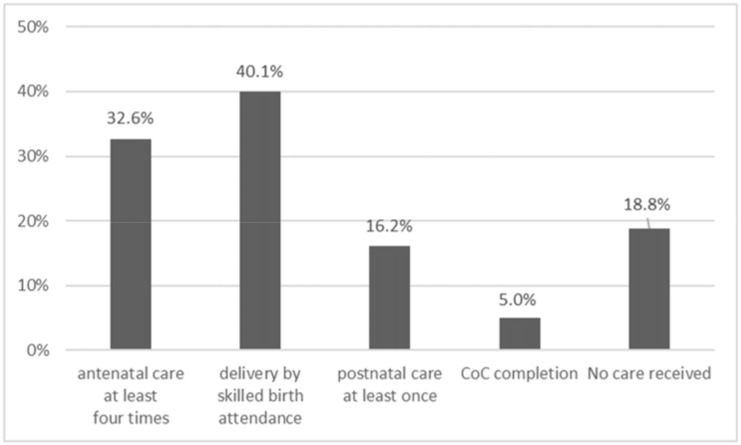
Rates of components of continuum of care availed by participants.

The frequency of women who availed the different components of continuum of care is shown in Fig 1. Facility delivery had the highest frequency, at 40.1%. Only 5.0% of the women included in the study completed all the necessary care visits. In contrast, 18.8% did not receive any care.

**Fig 2:**
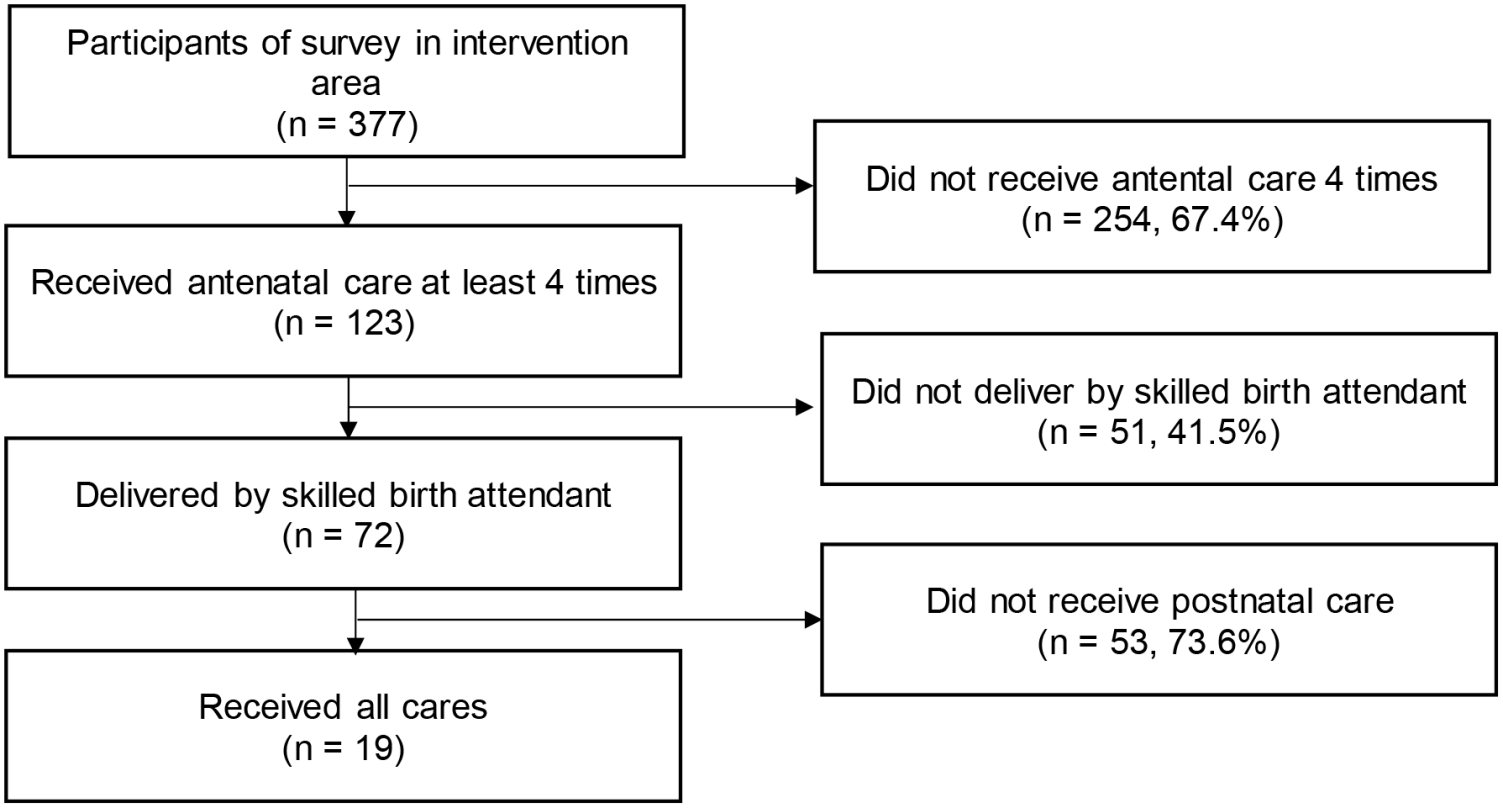
Flowchart of discontinuation from continuum care.

The numbers and percentage of women who discontinued the continuum of care from each stage of the continuum of care are shown in Fig 2. The highest discontinuation rate was at the postnatal care stage (73.6%), while the lowest was at the delivery stage (41.5%).

The characteristics of women who completed different stages of care are shown in Table 1. Nineteen women (5.0%) accomplished all stages of the continuum of care. The literacy level, husband’s education, ethnicity, actual distance to health center, knowledge on antenatal care, and knowledge on postnatal care significantly differed among women who accomplished the continuum of care compared to those who did not.

Fifty-three women (14.1%) discontinued from continuum of care at the postnatal stage. Their literacy levels, neonatal complication experiences, and knowledge on postnatal care significantly differed from those of the other participants.

Forty-one women (10.9%) discontinued the continuum of care at the delivery stage. Their age, literacy levels, husband’s education, number of children, neonatal complication experiences, and distance of their village to the health center significantly differed from those of the other participants.

Overall, 71 women (18.8%) discontinued the continuum of care at the antenatal care stage, indicating they did not receive any care. Compared to those of the other participants, their literacy levels, husband’s education, occupations, ethnicity, number of children, neonatal complication experiences, distance of their village to the health center, and knowledge on antenatal care, facility delivery, and postnatal care significantly differed.

### Factors associated with the completion of the continuum of care

The factors associated with the completion of the continuum of care among women are presented in Table 2. Accomplishing all the stages of care was negatively associated with a long distance to the health center (adjusted odds ratio [AOR]: 0.877; 95% confidence interval [CI]: 0.791–0.972).

**Table 2:** Factors associated with the completion of the continuum of care.

	a) Accomplished all cares (vs. not accomplished) n = 377		
Antenatal care at least four times	Yes		
Skilled birth attendance	Yes		
Postnatal care at least once	Yes		
	Adjusted odds ratio	Confidence Interval	
Age (year)	0.998	0.914	1.090
Literacy			
Not able to read	(Ref)		
Able to read	3.545	0.955	13.152
Husband’s education			
No education	(Ref)		
Official education	1.947	0.473	8.024
Socioeconomic status			
Low or lower middle	(Ref)		
Upper middle or high	1.373	0.436	4.326
Ethnicity			
Khmer	(Ref)		
Other	1.747	0.347	8.805
Charay	0.396	0.083	1.901
Number of children	1.064	0.632	1.794
Child’s age (month)	1.049	0.970	1.136
Child’s gender (female)	1.615	0.510	5.117
Child had health problem within 6 weeks after birth (yes)	0.578	0.168	1.984
Actual distance to health center (km)	0.877	0.791	0.972*

### Factors associated with discontinuation from the continuum of care

In Table 3, factors associated with different categories of women’s discontinuation from the continuum of care can be viewed. Awareness regarding postnatal care was negatively associated with women who discontinued the continuum of care during the postnatal stage (AOR: 0.008; 95% CI: 0.001–0.086).

**Table 3:** Factors associated with discontinuation from the continuum of care.

Discontinuation levels	b) Discontinued at postnatal care stage (vs. accomplished all cares) n = 72			c) Discontinued at delivery stage (vs. received antenatal care and did not discontinue at delivery stage) n = 113			d) Discontinued at antenatal care stage/received no care (vs. received any care) n = 377		
Antenatal care at least four times	Yes			Yes			No		
Skilled birth attendance	Yes			No			No		
Postnatal care at least once	No			No			No		
	Adjusted odds ratio	Confidence Interval		Adjusted odds ratio	Confidence Interval		Adjusted odds ratio	Confidence Interval	
Age (year)	0.954	0.776	1.173	0.940	0.833	1.061	1.026	0.967	1.089
Literacy									
Not able to read	(Ref)			(Ref)			(Ref)		
Able to read	0.145	0.014	1.480	0.269	0.079	0.910*	0.348	0.125	0.968*
Husband’s education									
No education	(Ref)			(Ref)			(Ref)		
Official education	1.055	0.103	10.853	0.943	0.287	3.104	0.248	0.111	0.551**
Socioeconomic status									
Low or lower middle	(Ref)			(Ref)			(Ref)		
Upper middle or high	0.149	0.018	1.235	0.403	0.125	1.299	1.171	0.570	2.407
Ethnicity									
Khmer	(Ref)			(Ref)			(Ref)		
Other	0.269	0.014	5.104	0.395	0.081	1.931	-	-	-
Charay	0.904	0.108	7.559	0.407	0.096	1.722	-	-	-
Number of children	0.666	0.280	1.584	1.679	0.968	2.911	1.011	0.763	1.340
Child’s age (month)	0.926	0.803	1.067	0.990	0.921	1.065	0.988	0.939	1.039
Child’s gender (female)	0.473	0.056	4.014	1.155	0.411	3.246	1.137	0.562	2.300
Child had health problem within 6 weeks after birth	-	-	-	0.286	0.090	0.904*	3.075	1.310	7.215**
Actual distance to health center (km)	1.053	0.907	1.224	1.103	1.019	1.193*	1.054	1.012	1.099*
Knowledge									
Know antenatal care is recommended	-	-	-	-	-	-	0.158	0.077	0.325**
Know facility delivery is recommended	-	-	-	0.950	0.159	5.677	0.123	0.051	0.297**
Know postnatal care is recommended	0.008	0.001	0.086**	0.653	0.173	2.468	0.630	0.197	2.012

* <0.05;

**<0.01

When compared to women who could not read, those who are able to read were less likely to discontinue at the delivery stage (AOR: 0.269; 95% CI: 0.079–0.910). Those living far from the health center were also more likely to discontinue (AOR: 1.103; 95% CI: 1.019–1.193). Meanwhile, women with children with health problems within 6 weeks of birth were less likely to discontinue (AOR: 0.286; 95% CI: 0.090–0.904).

Regarding factors associated with women who discontinued the continuum of care at the antenatal care stage (received any of the necessary care), compared to women who could not read, those who were able to read were less likely to discontinue (AOR: 0.348; 95% CI: 0.125–0.968). Having a husband with an official education was negatively associated with not receiving care when compared to having a husband with no education (AOR: 0.248; 95% CI: 0.111–0.551). Women who had children with health problems within 6 weeks after birth (AOR: 3.075; 95% CI: 1.310–7.215) or living in an area farther from the health center (AOR: 1.054; 95% CI: 1.012–1.099) were more likely to discontinue. Awareness regarding antenatal care (AOR: 0.158; 95% CI: 0.077–0.325) and on facility delivery (AOR: 0.123; 95% CI: 0.051–0.297) were negatively associated with discontinuation.

## Discussion

This study is among the few studies that have assessed the completion of the continuum of care, the discontinuation rate, and their associated factors among women in Ratanakiri, Cambodia. In this study, we demonstrated new and important findings. First, only 5.0% of women completed the continuum of care. Meanwhile, 18.8% of the women did not receive any care during pregnancy, delivery, and after birth. Second, the discontinuation rate was the highest at the postnatal care stage compared to other care stages. Third, not receiving any care was associated with a higher frequency of neonatal complications within 6 weeks of birth. Furthermore, the distance from the village to the health facility influenced the women’s completion of the continuum of care.

This study demonstrated that the completion rate for the continuum of care was only 5.0%. This was lower than that reported in a previous study in Cambodia conducted by Wang et al., which analyzed the completion rate based on the Demographic and Health Survey data. In that study, the overall rate of completion for the continuum of care in the Ratanakiri/Mondulkiri provinces was 24.0% [20]. The significantly lower rate of completion in our study compared with that of Wang’s study may be attributed to the inclusion of women who received antenatal care at least once in Wang’s study, while our study included only those who received antenatal care at least four times. However, the relatively low completion rate suggests that most pregnant women and newborns who lived in our study areas were unaware of their health status and were at risk of maternal and neonatal complications.

This study demonstrated that 18.8% of women in Ratanakiri Province did not receive any care. These findings indicate the need for further efforts to improve not only the completion rate of the continuum of care, but also to reduce the number of women who do not receive any care. Furthermore, in this study, receiving no care was associated with neonatal complications. Thus, receiving no care could significantly impact a newborn’s health. Additionally, the lack of knowledge on antenatal care or facility delivery was associated with receiving no care. Promoting the necessary care during pregnancy and delivery would be a key component to improve the rate of availing care.

The discontinuation rate was highest during the postnatal care stage, and the rate of availed postnatal care was the lowest among other components of the continuum of care. This finding also differed from that in Wang’s study, in which women who delivered through a skilled birth attendant were more likely to receive postnatal care [20]. This could be due to the misreport of mothers mentioned in the limitations of their study. In that study, participating mothers could have confused care that was part of delivery care as postnatal care, and this might have increased the rate of the mother’s high adherence to postnatal care after delivery. In our study, postnatal care was considered separate from delivery care; thus, the high discontinuation rate in this study is more reflective of the real level of care availed by the mother. The World Health Organization recommends postnatal newborn care within 48 hours, at 6–7 days, and 6 weeks after birth [33]. However, in this study, only few women received care during any of those stages, and none of them completed all stages of postnatal care. These data suggest that necessary care for complications might be delayed among newborns. Women must be encouraged and promoted by health workers to avail postnatal care once they received antenatal care and to deliver assisted by a skilled birth attendant.

In this study, women’s discontinuation from the continuum of care at the delivery stage was negatively associated with neonatal complications. Our finding that discontinuation from care restrains neonatal complications, seems to be contradicting. However, these results may imply that receiving antenatal care at least helps reduce the incidence of neonatal complications. This could be because antenatal care is an entry point in the continuum of care. Women are recommended to deliver with assistance from a skilled birth attendant and receive postnatal care through the sensitization during the antenatal period [34, 35]. Thus, receiving antenatal care and health education on the continuum of care may increase the women’s attention to preventing health problems.

Distance to the health facility was associated with the completion of the continuum of care. This finding is consistent with that of previous studies [26, 36]. Our study site has rather severe geographical and infrastructural challenges; paved roads are limited, and the roads become often unavailable for several months during the rainy season. In particular, delivery at night or vehicle unavailability can discourage women from going to the health facility [36]. These women may be discouraged to go to a health facility due to their limited access to health care services [37]. As such, the distance to the health facility can be a key factor to receiving care in this study site. To address this problem, a village-based health care system, such as a telemedicine system that provides basic antenatal care or postnatal care for women and newborns who live in remote areas, must be established. Existing village-based systems such as community health workers programs could be explored for use within maternal and child health programs. In the provinces of Cambodia, the village malaria worker system has been expanded since 2004 as a community health workers system specific for malaria. The village malaria workers are responsible for directly observing therapy of malaria, screening, treating, and referring people, including pregnant women, to health centers [38]. Integrating maternal and child health care into the village malaria worker system could be a probable framework.

This study was subject to several limitations. First, we selected health centers based on their accessibility throughout the year. Thus, this has a risk of selection bias. Second, the study participants were women who had children aged <2 years. Given that they were asked to recall their pregnancy experience as far back as to 2.5 years, recall bias was possible. However, such bias in neonatal complications was minimized by collecting data from the health worker records. Despite these limitations, this study reports on the new concepts of the continuum of care and its association with the newborn’s health status. To the best of our knowledge, such studies have been rarely performed in this field, and these results can be used as a basis for developing further interventions or for longitudinal studies to improve newborn health.

## Conclusions

This study demonstrated that the completion rate of the continuum of care was 5.0% among women in Ratanakiri, Cambodia. Postnatal care could be a key to decreasing mother’s discontinuation from the continuum of care. To achieve this, postnatal care needs to be promoted during the antenatal period and at delivery.

This study indicated the need for efforts to reduce the number of women who do not receive any care to avoid neonatal complications. Furthermore, given that the long distance to the health facility was a barrier to the continuum of care, this study suggested a need for establishing a village-based health care system for use within maternal and child health programs to mitigate the inaccessibility to service.

## Supporting information

S1 File Code book. (XLSX)

S2 File Data set. (XLSX)
